# 2-(10-Phenyl­anthracen-9-yl)-2,3-di­hydro-1*H*-naphtho­[1,8-*de*][1,3,2]di­aza­borinine

**DOI:** 10.1107/S2414314625005759

**Published:** 2025-07-01

**Authors:** Fanyi Meng, Shimin Zhou

**Affiliations:** a Shandong Experimental High School, Jinan, 250001, People’s Republic of China; b Shandong Shanke Institute for Industrial Development Research Jinan 250022 People’s Republic of China; University of Aberdeen, United Kingdom

**Keywords:** crystal structure, anthracene, B(dan)

## Abstract

In the title compound, C_30_H_21_BN_2_, the dihedral angle between the naphtho­[1,8-*de*][1,3,2]di­aza­borinine moiety and the anthracene ring system is 79.30 (3)°. In the crystal, weak C—H⋯π inter­actions link the mol­ecules into a three-dimensional network.

## Abstract 2

A naphtho­[1,8-*de*][1,3,2]-di­aza­borinine-substituted anthracene structure is reported.

## Graphical abstract

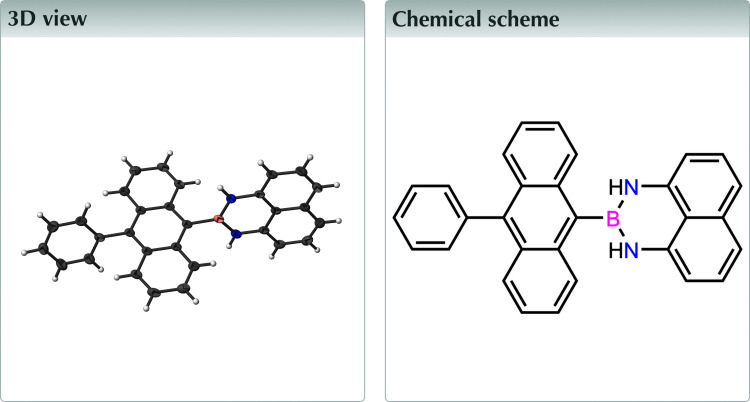

## Structure description

Aryl-B(dan) derivatives, where B(dan) is the naphtho­[1,8-*de*][1,3,2]di­aza­borinine fragment have emerged as not only stable boron-containing regents for coupling reactions (Yoshida *et al.*, 2020 ▸), chemosensors for explosives (Wan *et al.*, 2018 ▸) and per­oxy­nitrite species (Yoon *et al.*, 2024 ▸), but also precursors for large NBN-embedded polycyclic aromatic hydro­carbons (Ju *et al.*, 2021 ▸). Anthracene is a classical polycyclic aromatic hydro­carbon and a promising platform for optoelectronic materials; 9,10-di­phenyl­anthracene is well known as a reference of determining fluorescence quantum yield. Herein, we describe the synthesis and structure of the title compound, C_30_H_21_BN_2_, which is new derivative of a B(dan)-substituted anthracene.

**Figure 1:**
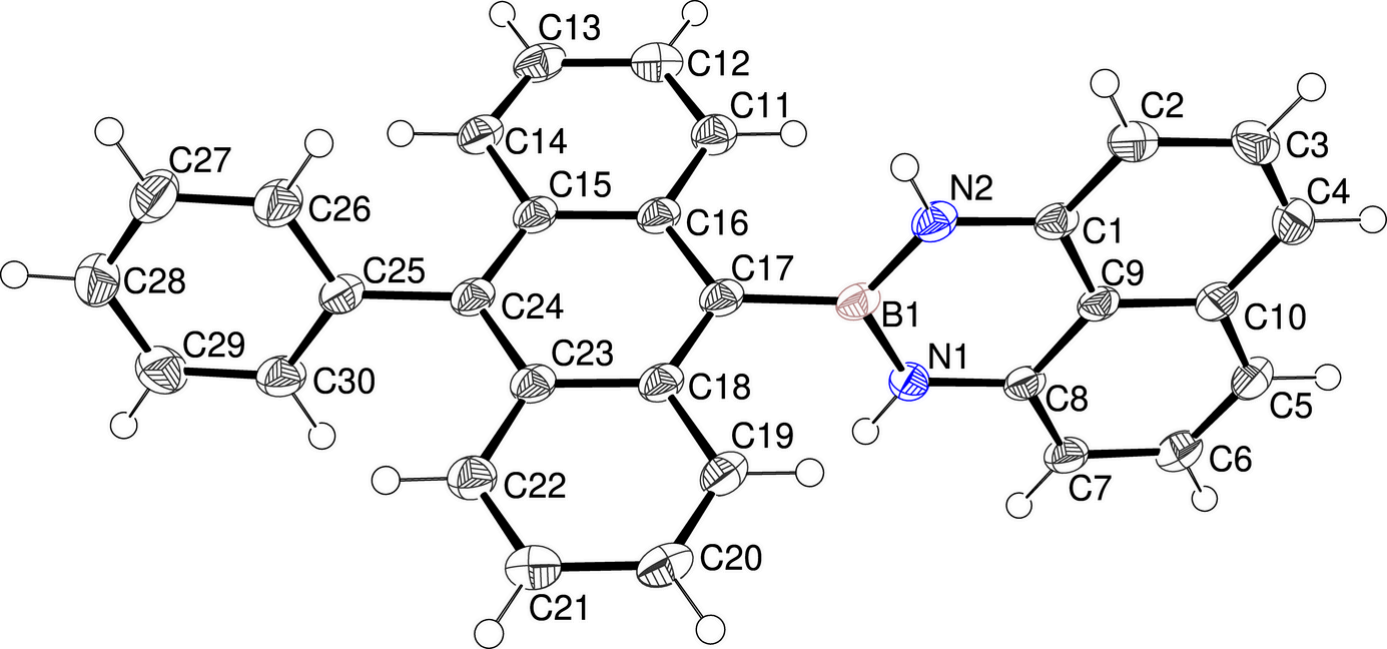
The mol­ecular structure of the title compound showing 50% displacement ellipsoids.

**Figure 2:**
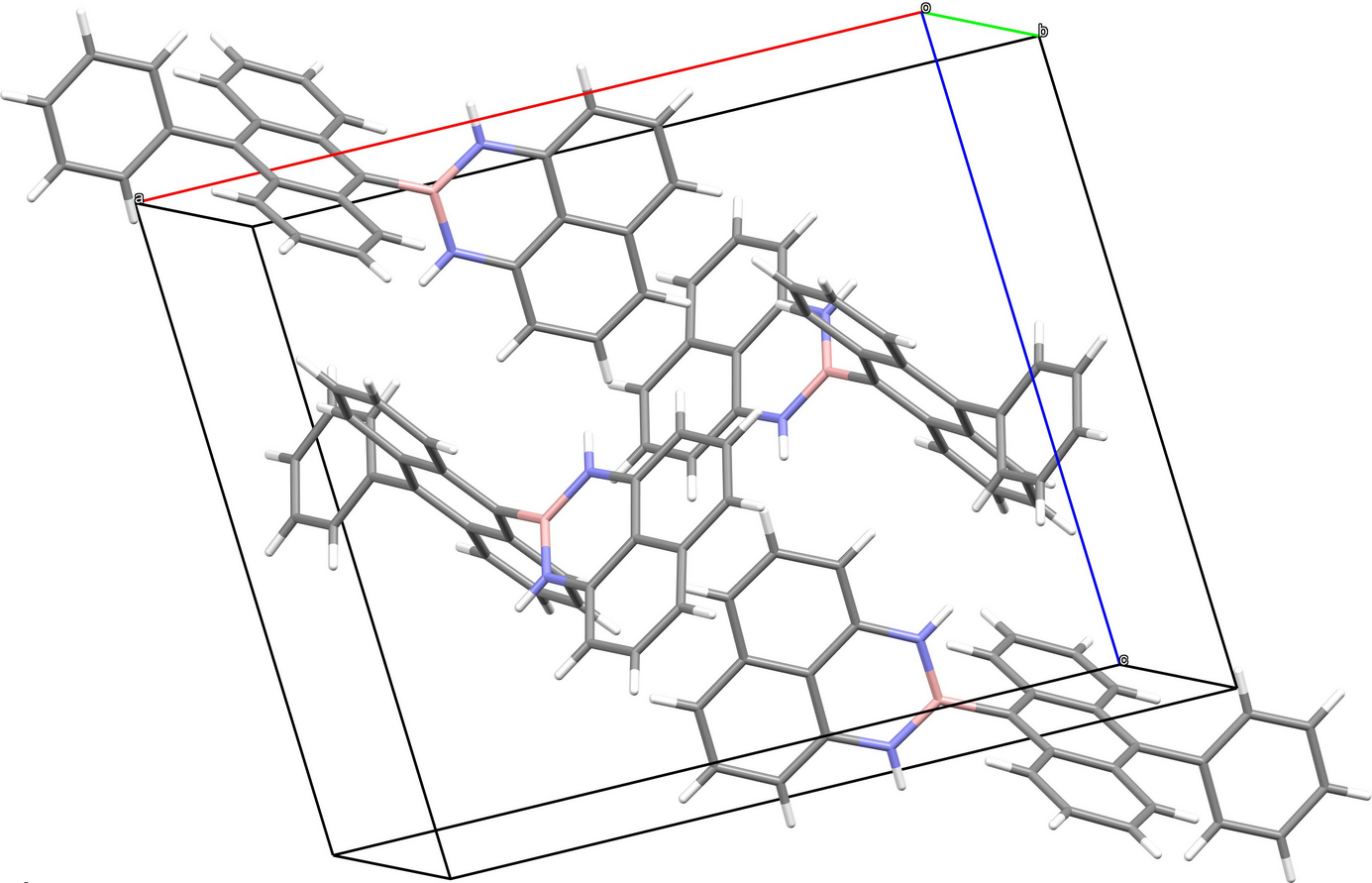
The unit-cell packing of the title compound viewed approximately down [001].

The title compound crystallizes in the monoclinic space group *P*2_1_/*c*. The C1–C10/N1/N2/B1 B(dan) moiety is close to planar, with the largest deviation from the least squares plane being −0.041 (1) Å for atom N2. Both the B(dan) unit at the 9-position and the C25–C30 phenyl ring at the 10-position are close to perpendicular to the central C11–C24 anthracene ring system with dihedral angles of 79.30 (3)° and 71.71 (6)°, respectively (Fig. 1 ▸). The lengths of the B—N bonds [1.4186 (19) and 1.412 (2) Å] are close to that of a localized B=N double bond (1.40 Å) and much shorter than that of a typical B—N single bond (1.68 Å), which indicates a significant π-electron delocalization in the NBN moiety. As shown in the packing diagram (Fig. 2 ▸), weak C—H⋯π inter­actions (Table 1 ▸) link the mol­ecules into a three-dimensional network.

## Synthesis and crystallization

The synthesis of 9-B(dan)-10-phenyl-anthracene followed the previously reported procedure (Wan *et al.*, 2018 ▸). 9-B(OH)2–10-phenyl-anthracene, 1,8-di­amine-naphthalene and anhydrous magnesium sulfate was added into adequate toluene. After heating 12 h at 120°C, the mixture was cooled to room temperature and concentrated under vacuum then purified by column chromatography of silica gel.

Single crystals of the title compound were obtained as pale-yellow plates by slow diffusion of hexane into its chloro­form solution at room temperature. A suitable crystal for collection was chosen under an optical microscope and quickly coated with high vacuum grease (Dow Corning Corporation) to prevent decomposition.

## Refinement

Crystal data, data collection and structure refinement details are summarized in Table 2 ▸.

## Supplementary Material

Crystal structure: contains datablock(s) global, I. DOI: 10.1107/S2414314625005759/hb4524sup1.cif

Structure factors: contains datablock(s) I. DOI: 10.1107/S2414314625005759/hb4524Isup2.hkl

Supporting information file. DOI: 10.1107/S2414314625005759/hb4524Isup3.cml

CCDC reference: 2467118

Additional supporting information:  crystallographic information; 3D view; checkCIF report

## Figures and Tables

**Table 1:** Hydrogen-bond geometry (Å, °) *Cg*1, *Cg*3, *Cg*4 and *Cg*6 are the centroids of the C1/C8/C9/N1/N2/B1, C5–C10, C11–C16 and C18–C23 rings, respectively.

*D*—H⋯*A*	*D*—H	H⋯*A*	*D*⋯*A*	*D*—H⋯*A*
C2—H2⋯*Cg*6^i^	0.95	2.86	3.7466 (17)	156
C3—H3⋯*Cg*3^ii^	0.95	2.85	3.4906 (17)	125
C4—H4⋯*Cg*1^ii^	0.95	2.67	3.5538 (18)	156
C6—H6⋯*Cg*4^iii^	0.95	2.83	3.6552 (17)	146
C27—H27⋯*Cg*6^iv^	0.95	2.73	3.5364 (18)	143

Symmetry codes: (i) 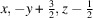; (ii) 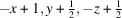; (iii) 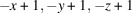; (iv) 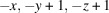.

**Table 2:** Experimental details

Crystal data	
Chemical formula	C_30_H_21_BN_2_
*M*_r_	420.30
Crystal system, space group	Monoclinic, *P*2_1_/*c*
Temperature (K)	150
*a*, *b*, *c* (Å)	17.5431 (7), 8.6734 (3), 14.3967 (5)
β (°)	95.268 (2)
*V* (Å^3^)	2181.32 (14)
*Z*	4
Radiation type	Cu *K*α
μ (mm^−1^)	0.57
Crystal size (mm)	0.60 × 0.30 × 0.03

Data collection	
Diffractometer	Bruker APEXII CCD
Absorption correction	Multi-scan (*SADABS*; Krause *et al.*, 2015 ▸)
*T*_min_, *T*_max_	0.727, 0.983
No. of measured, independent and observed [*I* > 2σ(*I*)] reflections	21224, 4302, 3582
*R*_int_	0.075
(sin θ/λ)_max_ (Å^−1^)	0.618

Refinement	
*R*[*F*^2^ > 2σ(*F*^2^)], *wR*(*F*^2^), *S*	0.066, 0.191, 1.06
No. of reflections	4302
No. of parameters	299
H-atom treatment	H-atom parameters constrained
Δρ_max_, Δρ_min_ (e Å^−3^)	0.31, −0.46

Computer programs: *APEX2* and *SAINT* (Bruker, 2014 ▸), *SHELXS97* (Sheldrick, 2008 ▸), *SHELXL2014/7* (Sheldrick, 2015 ▸) and *SHELXTL* (Sheldrick, 2008 ▸).

## full crystallographic data

### 2-(10-Phenylanthracen-9-yl)-2,3-dihydro-1H-naphtho[1,8-de][1,3,2]diazaborinine Crystal data

**Table:**

C_30_H_21_BN_2_	*F*(000) = 880
*M**_r_* = 420.30	*D*_x_ = 1.280 Mg m^−^^3^
Monoclinic, *P*2_1_/*c*	Cu *K*α radiation, λ = 1.54178 Å
*a* = 17.5431 (7) Å	Cell parameters from 8257 reflections
*b* = 8.6734 (3) Å	θ = 2.5–72.4°
*c* = 14.3967 (5) Å	µ = 0.57 mm^−^^1^
β = 95.268 (2)°	*T* = 150 K
*V* = 2181.32 (14) Å^3^	Plate, yellow
*Z* = 4	0.60 × 0.30 × 0.03 mm

### 2-(10-Phenylanthracen-9-yl)-2,3-dihydro-1H-naphtho[1,8-de][1,3,2]diazaborinine Data collection

**Table:**

Bruker APEXII CCD diffractometer	3582 reflections with *I* > 2σ(*I*)
Detector resolution: 8.3 pixels mm^-1^	*R*_int_ = 0.075
φ and ω scans	θ_max_ = 72.4°, θ_min_ = 2.5°
Absorption correction: multi-scan (SADABS; Krause *et al.*, 2015)	*h* = −21→21
*T*_min_ = 0.727, *T*_max_ = 0.983	*k* = −10→8
21224 measured reflections	*l* = −17→17
4302 independent reflections	

### 2-(10-Phenylanthracen-9-yl)-2,3-dihydro-1H-naphtho[1,8-de][1,3,2]diazaborinine Refinement

**Table:**

Refinement on *F*^2^	Hydrogen site location: inferred from neighbouring sites
Least-squares matrix: full	H-atom parameters constrained
*R*[*F*^2^ > 2σ(*F*^2^)] = 0.066	*w* = 1/[σ^2^(*F*_o_^2^) + (0.1385*P*)^2^ + 0.0802*P*] where *P* = (*F*_o_^2^ + 2*F*_c_^2^)/3
*wR*(*F*^2^) = 0.191	(Δ/σ)_max_ < 0.001
*S* = 1.06	Δρ_max_ = 0.31 e Å^−^^3^
4302 reflections	Δρ_min_ = −0.46 e Å^−^^3^
299 parameters	Extinction correction: *SHELXL2014/7* (Sheldrick, 2015), Fc^*^=kFc[1+0.001xFc^2^λ^3^/sin(2θ)]^-1/4^
0 restraints	Extinction coefficient: 0.0049 (10)
Primary atom site location: structure-invariant direct methods	

### 2-(10-Phenylanthracen-9-yl)-2,3-dihydro-1H-naphtho[1,8-de][1,3,2]diazaborinine Special details

**Table:**

Geometry. All esds (except the esd in the dihedral angle between two l.s. planes) are estimated using the full covariance matrix. The cell esds are taken into account individually in the estimation of esds in distances, angles and torsion angles; correlations between esds in cell parameters are only used when they are defined by crystal symmetry. An approximate (isotropic) treatment of cell esds is used for estimating esds involving l.s. planes.

### 2-(10-Phenylanthracen-9-yl)-2,3-dihydro-1H-naphtho[1,8-de][1,3,2]diazaborinine Fractional atomic coordinates and isotropic or equivalent isotropic displacement parameters (Å^2^)

**Table:**

	*x*	*y*	*z*	*U*_iso_*/*U*_eq_	
N1	0.40392 (7)	0.71226 (15)	0.48003 (8)	0.0253 (3)	
H1n	0.4102	0.6708	0.5360	0.030*	
N2	0.32303 (7)	0.77065 (15)	0.34048 (8)	0.0258 (3)	
H2n	0.2787	0.7630	0.3069	0.031*	
C1	0.38142 (8)	0.85573 (17)	0.30400 (10)	0.0229 (3)	
C2	0.37099 (9)	0.92903 (18)	0.21940 (10)	0.0283 (4)	
H2	0.3225	0.9253	0.1843	0.034*	
C3	0.43185 (10)	1.00993 (18)	0.18426 (11)	0.0318 (4)	
H3	0.4237	1.0623	0.1262	0.038*	
C4	0.50265 (10)	1.01356 (19)	0.23321 (11)	0.0309 (4)	
H4	0.5435	1.0659	0.2078	0.037*	
C5	0.58784 (9)	0.9433 (2)	0.37404 (12)	0.0315 (4)	
H5	0.6299	0.9936	0.3500	0.038*	
C6	0.59746 (9)	0.8743 (2)	0.45948 (11)	0.0319 (4)	
H6	0.6461	0.8783	0.4943	0.038*	
C7	0.53651 (9)	0.79721 (19)	0.49716 (10)	0.0275 (4)	
H7	0.5442	0.7503	0.5569	0.033*	
C8	0.46576 (8)	0.79027 (17)	0.44684 (10)	0.0233 (3)	
C9	0.45398 (8)	0.86217 (16)	0.35783 (10)	0.0226 (3)	
C10	0.51564 (9)	0.94070 (18)	0.32070 (10)	0.0264 (4)	
C11	0.29367 (8)	0.38239 (19)	0.36154 (10)	0.0285 (4)	
H11	0.3309	0.4444	0.3354	0.034*	
C12	0.28219 (9)	0.2357 (2)	0.33022 (11)	0.0318 (4)	
H12	0.3111	0.1964	0.2828	0.038*	
C13	0.22677 (9)	0.14105 (19)	0.36864 (11)	0.0313 (4)	
H13	0.2192	0.0382	0.3470	0.038*	
C14	0.18461 (9)	0.19629 (19)	0.43598 (10)	0.0282 (4)	
H14	0.1479	0.1311	0.4607	0.034*	
C15	0.19434 (8)	0.35107 (18)	0.47053 (10)	0.0245 (4)	
C16	0.25144 (8)	0.44634 (18)	0.43274 (10)	0.0241 (3)	
C17	0.26557 (8)	0.59722 (18)	0.46628 (10)	0.0242 (4)	
C18	0.22333 (8)	0.65395 (18)	0.53736 (10)	0.0246 (4)	
C19	0.23519 (9)	0.80720 (19)	0.57303 (11)	0.0287 (4)	
H19	0.2711	0.8721	0.5466	0.034*	
C20	0.19669 (9)	0.8622 (2)	0.64341 (11)	0.0319 (4)	
H20	0.2066	0.9635	0.6665	0.038*	
C21	0.14117 (9)	0.7681 (2)	0.68292 (11)	0.0327 (4)	
H21	0.1142	0.8069	0.7322	0.039*	
C22	0.12693 (9)	0.6227 (2)	0.64991 (11)	0.0292 (4)	
H22	0.0896	0.5617	0.6766	0.035*	
C23	0.16661 (8)	0.55915 (18)	0.57620 (10)	0.0243 (4)	
C24	0.15212 (8)	0.40916 (18)	0.54149 (10)	0.0242 (4)	
C25	0.09206 (8)	0.31027 (18)	0.57894 (10)	0.0253 (4)	
C26	0.02449 (9)	0.2781 (2)	0.52390 (11)	0.0342 (4)	
H26	0.0157	0.3231	0.4637	0.041*	
C27	−0.03026 (9)	0.1806 (2)	0.55622 (13)	0.0394 (4)	
H27	−0.0760	0.1593	0.5179	0.047*	
C28	−0.01857 (10)	0.1149 (2)	0.64349 (13)	0.0382 (4)	
H28	−0.0558	0.0472	0.6650	0.046*	
C29	0.04799 (11)	0.1482 (2)	0.69986 (12)	0.0382 (4)	
H29	0.0561	0.1047	0.7605	0.046*	
C30	0.10270 (9)	0.2451 (2)	0.66742 (10)	0.0316 (4)	
H30	0.1481	0.2672	0.7063	0.038*	
B1	0.33158 (10)	0.6966 (2)	0.42817 (12)	0.0240 (4)	

### 2-(10-Phenylanthracen-9-yl)-2,3-dihydro-1H-naphtho[1,8-de][1,3,2]diazaborinine Atomic displacement parameters (Å^2^)

**Table:**

	*U*^11^	*U*^22^	*U*^33^	*U*^12^	*U*^13^	*U*^23^
N1	0.0295 (6)	0.0258 (7)	0.0196 (6)	−0.0046 (5)	−0.0034 (5)	0.0037 (5)
N2	0.0264 (6)	0.0237 (7)	0.0257 (6)	−0.0020 (5)	−0.0062 (5)	0.0004 (5)
C1	0.0307 (7)	0.0151 (7)	0.0221 (7)	0.0003 (5)	−0.0025 (5)	−0.0026 (5)
C2	0.0397 (8)	0.0203 (8)	0.0234 (7)	0.0032 (6)	−0.0042 (6)	−0.0002 (6)
C3	0.0521 (9)	0.0205 (8)	0.0227 (7)	0.0015 (7)	0.0030 (6)	0.0035 (6)
C4	0.0426 (8)	0.0202 (8)	0.0306 (8)	−0.0042 (6)	0.0066 (6)	−0.0003 (6)
C5	0.0317 (7)	0.0285 (9)	0.0343 (8)	−0.0101 (6)	0.0031 (6)	−0.0058 (6)
C6	0.0282 (7)	0.0332 (10)	0.0329 (8)	−0.0060 (6)	−0.0039 (6)	−0.0057 (7)
C7	0.0315 (7)	0.0273 (9)	0.0225 (7)	−0.0036 (6)	−0.0052 (6)	−0.0017 (6)
C8	0.0287 (7)	0.0191 (8)	0.0214 (7)	−0.0024 (5)	−0.0016 (5)	−0.0040 (5)
C9	0.0302 (7)	0.0152 (8)	0.0219 (7)	−0.0012 (5)	−0.0009 (5)	−0.0043 (5)
C10	0.0349 (8)	0.0179 (8)	0.0263 (7)	−0.0028 (6)	0.0031 (6)	−0.0050 (6)
C11	0.0310 (7)	0.0274 (9)	0.0262 (8)	−0.0006 (6)	−0.0022 (6)	0.0001 (6)
C12	0.0371 (8)	0.0306 (9)	0.0267 (8)	0.0043 (7)	−0.0029 (6)	−0.0040 (6)
C13	0.0397 (8)	0.0206 (9)	0.0314 (8)	0.0004 (6)	−0.0079 (6)	−0.0044 (6)
C14	0.0313 (7)	0.0245 (9)	0.0269 (8)	−0.0040 (6)	−0.0071 (6)	0.0006 (6)
C15	0.0267 (7)	0.0232 (8)	0.0218 (7)	−0.0027 (6)	−0.0076 (5)	0.0019 (6)
C16	0.0265 (6)	0.0234 (8)	0.0211 (7)	−0.0020 (6)	−0.0058 (5)	0.0005 (6)
C17	0.0251 (6)	0.0241 (8)	0.0217 (7)	−0.0019 (6)	−0.0062 (5)	0.0004 (6)
C18	0.0256 (7)	0.0246 (8)	0.0219 (7)	−0.0027 (6)	−0.0063 (5)	0.0004 (6)
C19	0.0289 (7)	0.0255 (9)	0.0302 (8)	−0.0052 (6)	−0.0050 (6)	−0.0016 (6)
C20	0.0365 (8)	0.0242 (9)	0.0334 (8)	−0.0014 (7)	−0.0054 (6)	−0.0070 (6)
C21	0.0356 (8)	0.0338 (10)	0.0281 (8)	0.0010 (7)	0.0002 (6)	−0.0062 (7)
C22	0.0306 (7)	0.0308 (9)	0.0254 (7)	−0.0019 (6)	−0.0017 (6)	−0.0006 (6)
C23	0.0249 (6)	0.0256 (8)	0.0209 (7)	−0.0013 (6)	−0.0063 (5)	0.0014 (6)
C24	0.0243 (6)	0.0253 (8)	0.0212 (7)	−0.0027 (6)	−0.0073 (5)	0.0022 (6)
C25	0.0282 (7)	0.0220 (8)	0.0248 (7)	−0.0027 (6)	−0.0032 (6)	−0.0009 (6)
C26	0.0333 (8)	0.0380 (10)	0.0294 (8)	−0.0073 (7)	−0.0072 (6)	0.0072 (7)
C27	0.0311 (8)	0.0440 (11)	0.0418 (9)	−0.0119 (7)	−0.0042 (7)	0.0017 (8)
C28	0.0399 (9)	0.0350 (10)	0.0408 (9)	−0.0110 (7)	0.0101 (7)	−0.0023 (7)
C29	0.0504 (10)	0.0370 (10)	0.0273 (8)	−0.0049 (8)	0.0035 (7)	0.0049 (7)
C30	0.0363 (8)	0.0331 (9)	0.0239 (8)	−0.0041 (7)	−0.0048 (6)	0.0010 (6)
B1	0.0284 (7)	0.0184 (9)	0.0244 (8)	−0.0013 (6)	−0.0017 (6)	−0.0043 (6)

### 2-(10-Phenylanthracen-9-yl)-2,3-dihydro-1H-naphtho[1,8-de][1,3,2]diazaborinine Geometric parameters (Å, º)

**Table:**

N1—H1n	0.8800	C15—C14	1.436 (2)
N1—C8	1.3993 (19)	C15—C24	1.409 (2)
N1—B1	1.4186 (19)	C16—C11	1.431 (2)
N2—H2n	0.8800	C16—C15	1.443 (2)
N2—C1	1.4030 (19)	C17—C16	1.409 (2)
N2—B1	1.412 (2)	C17—C18	1.406 (2)
C1—C9	1.429 (2)	C18—C19	1.433 (2)
C2—C1	1.371 (2)	C18—C23	1.443 (2)
C2—H2	0.9500	C19—H19	0.9500
C3—C2	1.410 (2)	C19—C20	1.355 (2)
C3—H3	0.9500	C20—H20	0.9500
C4—C3	1.371 (2)	C20—C21	1.428 (2)
C4—H4	0.9500	C21—H21	0.9500
C4—C10	1.409 (2)	C21—C22	1.363 (2)
C5—H5	0.9500	C22—H22	0.9500
C5—C6	1.364 (2)	C22—C23	1.432 (2)
C5—C10	1.420 (2)	C24—C23	1.408 (2)
C6—H6	0.9500	C24—C25	1.497 (2)
C6—C7	1.411 (2)	C25—C26	1.392 (2)
C7—H7	0.9500	C25—C30	1.391 (2)
C7—C8	1.3799 (19)	C26—H26	0.9500
C8—C9	1.423 (2)	C26—C27	1.391 (2)
C10—C9	1.423 (2)	C27—H27	0.9500
C11—H11	0.9500	C27—C28	1.378 (3)
C11—C12	1.359 (2)	C28—H28	0.9500
C12—H12	0.9500	C28—C29	1.390 (3)
C12—C13	1.423 (2)	C29—H29	0.9500
C13—H13	0.9500	C30—C29	1.388 (2)
C13—C14	1.360 (2)	C30—H30	0.9500
C14—H14	0.9500	B1—C17	1.582 (2)
			
C8—N1—H1n	118.3	C24—C15—C16	119.87 (14)
C8—N1—B1	123.41 (12)	C11—C16—C15	118.11 (14)
B1—N1—H1n	118.3	C17—C16—C11	121.29 (14)
C1—N2—H2n	118.2	C17—C16—C15	120.60 (14)
C1—N2—B1	123.54 (12)	C16—C17—B1	119.92 (13)
B1—N2—H2n	118.2	C18—C17—C16	119.11 (14)
N2—C1—C9	117.52 (12)	C18—C17—B1	120.87 (13)
C2—C1—N2	122.27 (13)	C17—C18—C19	121.09 (14)
C2—C1—C9	120.20 (14)	C17—C18—C23	120.72 (14)
C1—C2—H2	119.8	C19—C18—C23	118.19 (14)
C1—C2—C3	120.39 (14)	C18—C19—H19	119.1
C3—C2—H2	119.8	C20—C19—C18	121.89 (15)
C2—C3—H3	119.8	C20—C19—H19	119.1
C4—C3—C2	120.48 (14)	C19—C20—H20	119.9
C4—C3—H3	119.8	C19—C20—C21	120.13 (15)
C3—C4—H4	119.5	C21—C20—H20	119.9
C3—C4—C10	120.91 (15)	C20—C21—H21	120.0
C10—C4—H4	119.5	C22—C21—C20	119.98 (15)
C6—C5—H5	119.6	C22—C21—H21	120.0
C6—C5—C10	120.74 (14)	C21—C22—H22	119.0
C10—C5—H5	119.6	C21—C22—C23	121.92 (15)
C5—C6—H6	119.4	C23—C22—H22	119.0
C5—C6—C7	121.27 (14)	C22—C23—C18	117.86 (14)
C7—C6—H6	119.4	C24—C23—C18	119.83 (14)
C6—C7—H7	120.1	C24—C23—C22	122.30 (14)
C8—C7—C6	119.71 (14)	C15—C24—C25	119.38 (14)
C8—C7—H7	120.1	C23—C24—C15	119.86 (13)
N1—C8—C9	117.80 (12)	C23—C24—C25	120.77 (13)
C7—C8—N1	122.01 (13)	C26—C25—C24	120.04 (13)
C7—C8—C9	120.19 (14)	C30—C25—C24	121.55 (13)
C8—C9—C1	121.30 (14)	C30—C25—C26	118.39 (14)
C8—C9—C10	119.70 (13)	C25—C26—H26	119.7
C10—C9—C1	119.00 (13)	C27—C26—C25	120.62 (15)
C4—C10—C5	122.65 (14)	C27—C26—H26	119.7
C4—C10—C9	118.97 (14)	C26—C27—H27	119.8
C5—C10—C9	118.39 (14)	C28—C27—C26	120.46 (15)
C12—C11—H11	119.0	C28—C27—H27	119.8
C12—C11—C16	122.02 (15)	C27—C28—H28	120.2
C16—C11—H11	119.0	C27—C28—C29	119.54 (16)
C11—C12—H12	120.1	C29—C28—H28	120.2
C11—C12—C13	119.81 (15)	C28—C29—H29	120.0
C13—C12—H12	120.1	C30—C29—C28	119.94 (15)
C12—C13—H13	119.7	C30—C29—H29	120.0
C14—C13—C12	120.66 (15)	C25—C30—H30	119.5
C14—C13—H13	119.7	C29—C30—C25	121.03 (14)
C13—C14—H14	119.3	C29—C30—H30	119.5
C13—C14—C15	121.42 (15)	N1—B1—C17	121.18 (13)
C15—C14—H14	119.3	N2—B1—N1	116.33 (14)
C14—C15—C16	117.98 (14)	N2—B1—C17	122.47 (13)
C24—C15—C14	122.12 (14)		
			
N1—C8—C9—C1	0.2 (2)	C16—C11—C12—C13	−0.1 (2)
N1—C8—C9—C10	−179.01 (13)	C16—C15—C14—C13	−1.1 (2)
N1—B1—C17—C16	−100.23 (17)	C16—C15—C24—C23	−0.9 (2)
N1—B1—C17—C18	76.1 (2)	C16—C15—C24—C25	178.91 (12)
N2—C1—C9—C8	−2.5 (2)	C16—C17—C18—C19	−179.38 (12)
N2—C1—C9—C10	176.75 (12)	C16—C17—C18—C23	0.4 (2)
N2—B1—C17—C16	77.95 (19)	C17—C16—C11—C12	178.19 (13)
N2—B1—C17—C18	−105.73 (17)	C17—C16—C15—C14	−177.68 (12)
C1—N2—B1—N1	0.1 (2)	C17—C16—C15—C24	0.1 (2)
C1—N2—B1—C17	−178.11 (13)	C17—C18—C19—C20	−177.97 (14)
C2—C1—C9—C8	178.45 (13)	C17—C18—C23—C22	178.55 (13)
C2—C1—C9—C10	−2.3 (2)	C17—C18—C23—C24	−1.3 (2)
C3—C2—C1—N2	−178.32 (14)	C18—C17—C16—C11	−179.02 (13)
C3—C2—C1—C9	0.7 (2)	C18—C17—C16—C15	0.1 (2)
C3—C4—C10—C5	−179.27 (15)	C18—C19—C20—C21	−1.4 (2)
C3—C4—C10—C9	0.2 (2)	C19—C18—C23—C22	−1.62 (19)
C4—C3—C2—C1	1.4 (2)	C19—C18—C23—C24	178.57 (12)
C4—C10—C9—C1	1.8 (2)	C19—C20—C21—C22	0.1 (2)
C4—C10—C9—C8	−178.90 (14)	C20—C21—C22—C23	0.4 (2)
C5—C6—C7—C8	0.3 (2)	C21—C22—C23—C18	0.4 (2)
C5—C10—C9—C1	−178.64 (13)	C21—C22—C23—C24	−179.83 (14)
C5—C10—C9—C8	0.6 (2)	C23—C18—C19—C20	2.2 (2)
C6—C5—C10—C4	178.42 (15)	C23—C24—C25—C26	109.45 (17)
C6—C5—C10—C9	−1.1 (2)	C23—C24—C25—C30	−72.4 (2)
C6—C7—C8—N1	178.50 (14)	C24—C15—C14—C13	−178.80 (13)
C6—C7—C8—C9	−0.8 (2)	C24—C25—C26—C27	177.03 (16)
C7—C8—C9—C1	179.54 (13)	C24—C25—C30—C29	−177.20 (16)
C7—C8—C9—C10	0.3 (2)	C25—C24—C23—C18	−178.35 (12)
C8—N1—B1—N2	−2.6 (2)	C25—C24—C23—C22	1.8 (2)
C8—N1—B1—C17	175.65 (13)	C25—C26—C27—C28	0.3 (3)
C10—C4—C3—C2	−1.9 (2)	C25—C30—C29—C28	0.1 (3)
C10—C5—C6—C7	0.6 (3)	C26—C25—C30—C29	1.0 (3)
C11—C12—C13—C14	0.5 (2)	C26—C27—C28—C29	0.9 (3)
C11—C16—C15—C14	1.50 (19)	C27—C28—C29—C30	−1.1 (3)
C11—C16—C15—C24	179.29 (12)	C30—C25—C26—C27	−1.2 (3)
C12—C13—C14—C15	0.0 (2)	B1—N1—C8—C7	−176.85 (15)
C14—C15—C24—C23	176.77 (12)	B1—N1—C8—C9	2.4 (2)
C14—C15—C24—C25	−3.4 (2)	B1—N2—C1—C2	−178.65 (14)
C15—C16—C11—C12	−1.0 (2)	B1—N2—C1—C9	2.3 (2)
C15—C24—C23—C18	1.5 (2)	B1—C17—C16—C11	−2.6 (2)
C15—C24—C23—C22	−178.31 (13)	B1—C17—C16—C15	176.51 (12)
C15—C24—C25—C26	−70.4 (2)	B1—C17—C18—C19	4.3 (2)
C15—C24—C25—C30	107.78 (17)	B1—C17—C18—C23	−175.91 (12)

### 2-(10-Phenylanthracen-9-yl)-2,3-dihydro-1H-naphtho[1,8-de][1,3,2]diazaborinine Hydrogen-bond geometry (Å, º)

*Cg*1, *Cg*3, *Cg*4 and *Cg*6 are the centroids of the C1/C8/C9/N1/N2/B1, C5–C10, C11–C16 and C18–C23 rings, respectively.

**Table:**

*D*—H···*A*	*D*—H	H···*A*	*D*···*A*	*D*—H···*A*
C2—H2···*Cg*6^i^	0.95	2.86	3.7466 (17)	156
C3—H3···*Cg*3^ii^	0.95	2.85	3.4906 (17)	125
C4—H4···*Cg*1^ii^	0.95	2.67	3.5538 (18)	156
C6—H6···*Cg*4^iii^	0.95	2.83	3.6552 (17)	146
C27—H27···*Cg*6^iv^	0.95	2.73	3.5364 (18)	143

Symmetry codes: (i) *x*, −*y*+3/2, *z*−1/2; (ii) −*x*+1, *y*+1/2, −*z*+1/2; (iii) −*x*+1, −*y*+1, −*z*+1; (iv) −*x*, −*y*+1, −*z*+1.
